# Comparison of peri-implant soft tissue and crestal bone status of dental implants placed in prediabetic, type 2 diabetic, and non-diabetic individuals: a retrospective cohort study

**DOI:** 10.1186/s40729-020-00255-1

**Published:** 2020-10-05

**Authors:** Abdullah Alshahrani, Modhi Al Deeb, Saad Alresayes, Sameer A. Mokeem, Nawwaf Al-Hamoudi, Osama Alghamdi, Fahim Vohra, Tariq Abduljabbar

**Affiliations:** 1 Department of Prosthetic Dental Science, College Of Dentistry, King Saud University, Riyadh, Saudi Arabia; 2 Department of Periodontics and Community Dentistry, King Saud University, Riyadh, Saudi Arabia; 3 Department of Oral and Maxillofacial Surgery, College Of Dentistry, King Saud University, Riyadh, Saudi Arabia

**Keywords:** Alveolar bone loss, Bleeding on probing, Narrow-diameter implants, Prediabetes, Probing depth, Type 2 diabetes mellitus

## Abstract

**Background:**

Clinicoradiographic status of narrow-diameter implants (NDIs) among patients with prediabetes and type 2 diabetes mellitus (DM) is scarce. The aim was to address the clinicoradiographic status of NDIs placed prediabetic, type 2 diabetic, and non-diabetic individuals. In this retrospective cohort study, patients having undergone oral rehabilitation with NDI were included. The participants were divided into the following: (a) patients with prediabetes; (b) patients with poorly controlled type 2 DM; (c) patients with well-controlled type 2 DM; and (d) normoglycemic individuals. Demographic data was collected. In all groups, peri-implant plaque index (PI), gingival index (GI), probing depth (PD), and mesiodistal CBL were measured in all groups. Information related to implant dimensions, surface characteristics, insertion torque, implant geometry, duration of NDI in function, and jaw location of NDI was also recorded. Data normality was assessed and group comparisons were performed. A probability value under 0.01 was considered statistically significant.

**Results:**

Eighty-three patients (20 patients had prediabetes, 22 with poorly controlled type 2 DM, 20 with well-controlled type 2 DM, and 20 self-reported non-diabetic individuals) were included. The mean HbA1c levels were significantly higher among patients with prediabetes (*P* < 0.01) and poorly controlled type 2 DM (*P* < 0.01) than patients with well-controlled type 2 DM and non-diabetic controls. Peri-implant PI, GI, PD, and mesiodistal CBL levels were significantly higher among patients with pre-diabetes (*P* < 0.01) and poorly controlled type 2 DM (*P* < 0.01) than patients with well-controlled type 2 DM and non-diabetic controls. Peri-implant PI, GI, PD, and mesiodistal CBL levels were significantly higher among patients with poorly controlled type 2 DM (*P* < 0.01) than patients with prediabetes.

**Conclusion:**

Chronic hyperglycemia increases the risk of peri-implant diseases around NDIs.

## Introduction

The narrow-diameter implants (NDIs) are gaining popularity in clinical implant dentistry and related research as the chances of osseous augmentation and other invasive surgical procedures that may lead to complications such as nerve damage, wound dehiscence, uncontrolled hemorrhage, graft failures, and infections are minimal compared with conventional dental implants (diameter ≥ 4 mm) [1–3]. In indexed literature, the precise definition of NDIs remains controversial; however, dental implants with a diameter of up to 3.5 mm are categorized as NDIs [2, 3]. In an experimental study on canine-models, Chang et al. [4] reported that NDIs osseointegrate in a manner to conventional dental implants. According to Klein et al. [3], NDIs demonstrate success rates of up to 100% and can remain functionally stable in posterior load-bearing areas in both jaws. Furthermore, results from a systematic review and meta-analysis, reported that the survival rates of NDIs and regular diameter implants are comparable [5].

Chronic hyperglycemia is manifested in patients with prediabetes and poorly controlled type 2 diabetes mellitus (DM) [6, 7], and is a risk factor of periodontal and peri-implant diseases [6–13]. In vitro studies [14, 15] have reported that the formation and accumulation of advanced glycation endproducts in gingival and systemic tissues is increased in patients with chronic hyperglycemic conditions. These end products interact with their receptors (receptors for advanced glycation endproducts [RAGE]) and this causes an increased formation of proinflammatory cytokines such as interleukin [IL] 1β and tumor necrosis factor alpha (TNF-α) [14, 15]. These pathophysiological mechanisms have been associated with an increased peri-implant soft tissue inflammation (clinically manifested as an increased gingival index [GI] and probing depth [PD]) and crestal bone loss (CBL) around dental implants. Results from an experimental study [16] on rats showed that an impaired glycemic status jeopardizes implant osseointegration and compromises implant stability. In the study by Alrabiah et al. [17], levels of AGEs in the peri-implant sulcular fluid were significantly higher among patients with prediabetes and poorly controlled type 2 DM compared with patients without DM. However, under optimal glycemic control, dental implants can demonstrate successful stability and osseointegration, which is similar to that observed in systemically healthy individuals [18]. In the present study, it is hypothesized that the soft-tissue inflammatory parameters and CBL around NDIs are compromised in patients with poorly controlled type 2 DM and prediabetes compared with patients with well-controlled type 2 DM and systemically healthy controls.

The aim of the present retrospective cohort study was to assess the peri-implant soft-tissue and CBL around NDIs placed in patients with prediabetes, poorly and well-controlled type 2 DM, and non-diabetic individuals.

## Methods

### Ethics approval and consent to participate

The study was designed, conducted, and reported following the Consolidation Standards of Reporting Trials statement. The present study was performed following guidelines recognized by the Declaration of Helsinki as revised in 2013 for experimentation involving human patients. Ethical approval was obtained from the ethics research committee of center for specialist dental practice and clinical research, Saudi Arabia (UDCRC/019-12). Withdrawal was inconsequential. Signing the consent form was mandatory for all individuals.

### Study design and recruitment of participants

The present study was based on a retrospective cohort design and was conducted among residents of Riyadh, Saudi Arabia. All participants were recruited from the outpatient department center for specialist dental practice and clinical research. Patients were given verbal and written information about the objectives and methods of the present study. All individuals were invited to ask questions. Individuals that agreed to participate in the present study were requested to read and sign a consent form. Prior to signing the consent form, participants were again invited to ask questions in case they had any. Inclusion standards were as follows: patients diagnosed with prediabetes [19] and type 2 DM [20], and self-reported systemically healthy individuals with normal hemoglobin A1c (HbA1c) levels, and individuals that agreed to have HbA1c levels measured (see supplemental STROBE checklist).

### Exclusion criteria

Pregnant and/or lactating women, edentulous patients, patients with periodontitis, self-reported smokeless tobacco (ST) chewers and tobacco smokers, and patients with other self-reported medical anomalies including but not limited to type 1 DM, HIV positive and patients diagnosed with AIDS, renal diseases, hepatic disorders, and cardiovascular diseases were eliminated. Moreover, NDIs placed in grafted sites and patients that declined to sign the consent form were excluded. From a radiographic perspective, X-rays in which implant-abutment junction (IAJ) was not clearly visible for technical reasons were eliminated. Furthermore, patients that reported to have undergone non-surgical and/or surgical periodontal/peri-implant interventions were excluded.

### Questionnaire

Using a structured questionnaire demographic information (age, gender, daily tooth brushing, and flossing habits; duration of prediabetes and type 2 DM; and family history of hyperglycemia) was recorded. The questionnaire also gathered information about the education status. All participants were inquired if their highest level of education was (a) undergraduate (up to high school); (b) college level; or (c) post-graduate level (masters and/or doctorate). The questionnaire was managed by one experienced co-investigator (*kappa* 0.94). Medical archives of the consenting patients were also explored to verify the duration of prediabetes and type 2 DM and their treatments recommended by healthcare providers.

### Hemoglobin A1c

The HbA1c levels were measured as described elsewhere [21, 22]. In summary, HbA1c levels were recorded by a blinded investigator (*kappa* 0.9) using a calibrated commercially available kit (QuoTest, EKF Diagnostics, Magdeburg, Germany). An HbA1c level ≤ 5.6% was deemed normal glycemic status and higher values reflected hyperglycemia [23, 24]. Based upon their hemoglobin A1c levels, participants were classified into 4 groups: (a) patients with prediabetes (HbA1c 5.5 to 6.4%) [19, 25]; (b) patients with poorly controlled type 2 DM [20] (HbA1c ≥ 6.5%) [24]; (c) patients with well-controlled type 2 DM (HbA1c < 5.7%) [20, 25]; and (d) self-reported systemically healthy individuals with normal glycemic levels (HbA1c < 5.7%) [25].

### Clinicoradiographic status

In all patients, peri-implant GI [26], PD [27], and plaque index (PI) [26] were measured by a skilled and standardized examiner (*kappa* 0.89). These measurements were performed on 6 surfaces per implant (midlingual/palatal, distolingual/palatal, mesiolingual/palatal, distobuccal, midbuccal, and mesiobuccal). The long-cone paralleling technique [28, 29] was employed to record intra-oral digital radiographs for all NDIs (intra-oral X-ray systems—NOMAD/Pro-2 Gendex-Hatfield, PA, USA). In all radiographs, CBL was demarcated as the vertical void from 2 mm under the IAJ to the crest of interdental bone [30]. The clinicoradiographic evaluations were performed between May and November 2019 by a trained and calibrated investigator (*kappa* score 0.91).

### Characteristics of NDIs

The length and diameter of all NDIs (Eztetic implants-Zimmer implants, Zimmer Biomet Dental, Palm Beach Gardens, FL, USA) were 11.5 mm and 3.2 mm, respectively. The NDIs were tapered with rough surfaces. All NDIs were platform switched and all prostheses were retained with cement-retained restorations. Data related to the duration of NDIs in function, the participants were retrieved from the patients’ records.

### Statistical assessment

The collected data was statistically assessed (SPSS Version 20, Chicago, IL, USA) and Microsoft Excel 2013. Intra-examiner reliability was determined using Cohen’s *kappa* score. A *kappa* score of at least 80% was considered acceptable. Peri-implant inflammatory parameters (PI, GI, PD, and CBL) were expressed as means ± standard deviations. The Shapiro-Wilk test, independent *t* test, analysis of variance, and Bonferroni post hoc adjustment were done for multiple comparisons. Probability values which were under 0.05 were classified as “significant.” Sample size estimation was performed on the basis of data obtained from a pilot investigation. Statistical power determined using a computer-based software (n/Query Advisor/6, StatisticalSolutions, MA, USA) indicated that enrolling 23 patients/group would give 95% power with *α* of 0.05.

## Results

### The study cohort

Eighty-three patients were included (20 patients had prediabetes, 22 with poorly controlled type 2 DM, 20 with well-controlled type 2 DM, and 20 self-reported non-diabetic individuals) were included. There was no statistically significant difference in age and gender among patients in all groups. The mean duration of hyperglycemia in patients with prediabetes, poorly controlled type 2 DM, and well-controlled type 2 DM was 2.9 ± 0.2, 3.3 ± 1.1, and 3.5 ± 0.8 years, respectively. The mean HbA1c levels were significantly higher among patients with prediabetes (*P* < 0.01) and poorly controlled type 2 DM (*P* < 0.01) compared with patients with well-controlled type 2 DM and non-diabetic controls. The mean HbA1c levels were significantly higher among patients with poorly controlled type 2 DM compared with patients with prediabetes (*P* < 0.01), well-controlled type 2 DM (*P* < 0.01), and non-diabetic controls (*P* < 0.01). A family history of diabetes was more often reported by patients with prediabetes (47.6%) and poorly controlled type 2 DM (50%) than patients with well-controlled type 2 DM (25%) than non-diabetic individuals (15%). Post-graduate level education status was more prevalent in patients with well-controlled type 2 DM and non-diabetic controls compared with patients with prediabetes and poorly controlled type 2 DM. Tooth brushing twice daily was more often reported by non-diabetic controls and patients well-controlled type 2 DM compared with patients with prediabetes and poorly controlled type 2 DM. Most of the individuals in all groups reported to brush their teeth once daily and none reported to have ever used a dental floss (Table 1). Table 2 shows the characteristics of the implants investigated.

**Table 1:** Demographics of patients with prediabetes and type 2 diabetes mellitus and normoglycemic individuals

Parameters	Patients with prediabetes	Patients with poorly controlled type 2 DM	Patients with well-controlled type 2 DM	Normoglycemic individuals
Number of individuals	25	25	25	25
Gender (male:female)	20:5	22:3	18:7	19:6
Overall age in years (mean ± SD)	53.5 ± 4.5 years	52.7 ± 4.1 years	48.6 ± 0.8	52.5 ± 4.4 years
Age of males in years (mean ± SD)	55.2 ± 3.3 years	54.6 ± 1.6 years	50.3 ± 1.2 years	54.8 ± 3.8 years
Age of females in years (mean ± SD)	50.6 ± 2.5 years	49.5 ± 0.8 years	45.5 ± 0.8 years	48.1 ± 2.1 years
HbA1c levels (mean ± SD)	6.5 ± 0.4 %	8.8 ± 0.2%	4.7 ± 0.3%	4.4 ± 0.2%
Duration of prediabetes in years (mean ± SD)	1.6 ± 0.2 years	NA	NA	NA
Duration of type 2 DM in years (mean ± SD)	NA	4.3 ± 0.5 years	3.7 ± 0.4 years	NA
Family history of hyperglycemia (*n*) (%)	13 (52%)	16 (64%)	5 (20%)	3 (12%)
Daily tooth brushing				
Once daily (*n*) (%)	17 (68%)	20 (80%)	12 (48%)	14 (56%)
Twice daily (*n*) (%)	8 (32%)	5 (20%)	13 (52%)	11 (44%)
Dental flossing (*n*) (%)	None	None	None	None

**Table 2:** Characteristics of the narrow-diameter implants placed in patients with prediabetes and type 2 diabetes mellitus and normoglycemic individuals

Implant features	Patients with prediabetes (*n* = 25)	Patients with poorly controlled type 2 DM (*n* = 25)	Patients with well-controlled type 2 DM (*n* = 25)	Normoglycemic individuals (*n* = 25)
Number of implants (maxilla: mandible)	25 (13: 12)	25 (15: 10)	25 (13: 12)	25 (14: 11)
Implant placement in relation to crestal bone	Bone level	Bone level	Bone level	Bone level
Duration of implants in function in years (mean ± SD)	5.2 ± 0.2 years	5.3 ± 0.2 years	5.3 ± 0.3 years	5.4 ± 0.2 years
Implant surface	Moderately rough	Moderately rough	Moderately rough	Moderately rough
Implant geometry	Platform switched	Platform switched	Platform switched	Platform switched
Insertion torque	30-35 Ncm	30-35 Ncm	30-35 Ncm	30-35 Ncm
Implant loading protocol	Delayed loading	Delayed loading	Delayed loading	Delayed loading
Implant prosthesis retention	Cement retained	Cement retained	Cement retained	Cement retained

Delayed loading: Implants were loaded 3 to 4 months after placement

### Peri-implant clinicoradiographic parameters

**Fig. 1:**
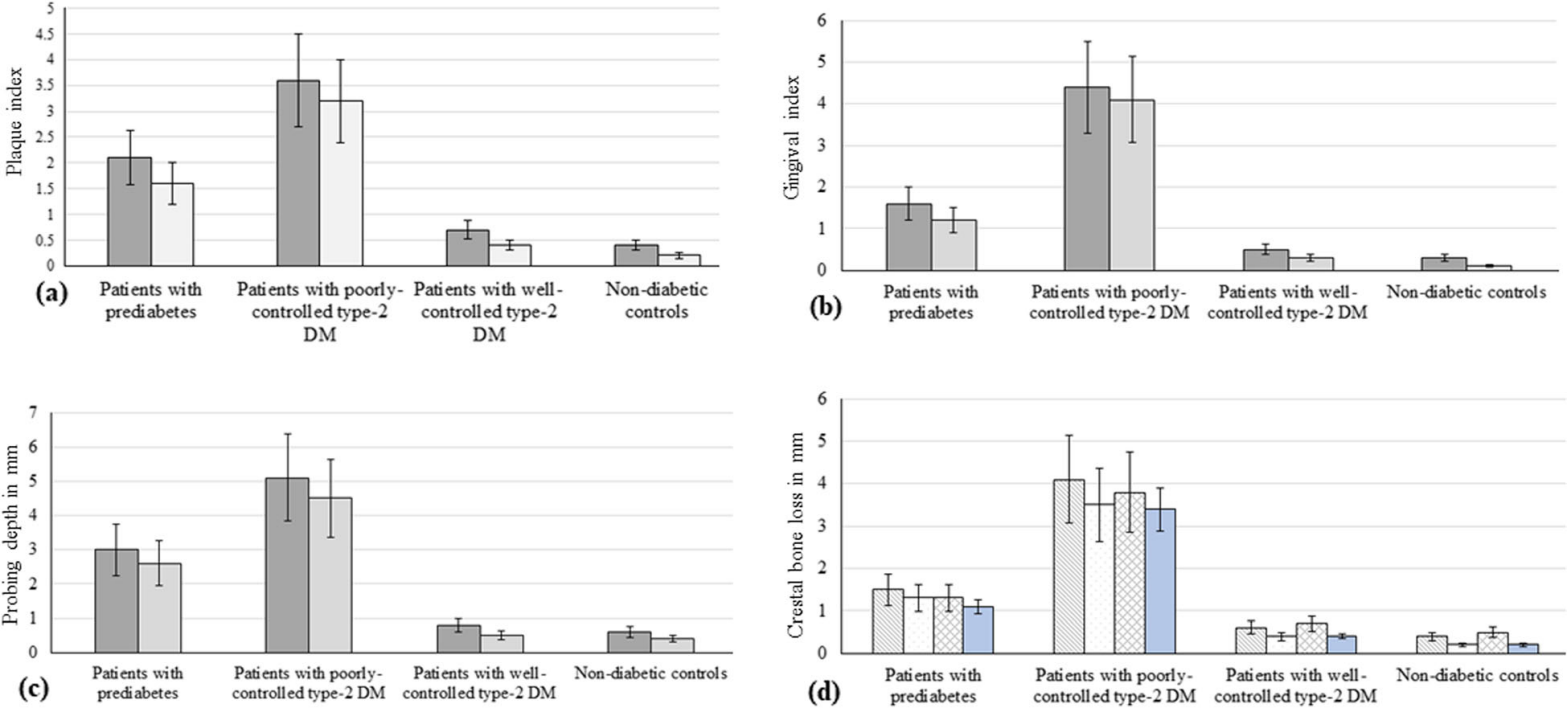
**a-d** Clinicoradiographic parameters around implants placed in the maxilla and mandible. Dark gray bars represent implants placed in the maxilla; light gray bars represent implants placed in the mandible; striped and dotted bars represent crestal bone loss on the mesial surface of implants placed in the maxilla and mandible, respectively; cross checked and blue bars represent crestal bone loss on the distal surface of implants placed in the maxilla and mandible, respectively

The peri-implant PI, GI, PD, and mesiodistal CBL levels were significantly higher among patients with pre-diabetes (*P* < 0.01) and poorly controlled type 2 DM (*P* < 0.01) compared with patients with well-controlled type 2 DM and non-diabetic controls. The peri-implant PI, GI, PD, and mesiodistal CBL levels were significantly higher among patients with poorly controlled type 2 DM (*P* < 0.01) compared with patients with prediabetes. There was no difference in PI, GI, PD, and mesiodistal CBL levels among patients with well-controlled type 2 DM and non-diabetic controls (Table 3). There was no influence of jaw location on clinicoradiographic parameters (Fig. 1a-d).

**Table 3:** Peri-implant gingival index, probing depth, and mesiodistal crestal bone loss among patients with prediabetes and type 2 diabetes mellitus and normoglycemic individuals

Peri-implant parameters	Patients with prediabetes	Patients with poorly controlled type 2 DM	Patients with well-controlled type 2 DM	Normoglycemic individuals
Plaque index (mean ± SD)	2.8 ± 0.2	3 ± 0.3	0.6 ± 0.2	0.4 ± 0.1
Gingival index (mean ± SD)	3 ± 0.2	3.3 ± 0.4	0.8 ± 0.2	0.5 ± 0.2
Probing depth (mean ± SD)	4.1 ± 0.3 mm*	4.5 ± 0.4 mm*	1.4 ± 0.2 mm	1.2 ± 0.3 mm
Crestal bone loss (mesial) in millimeter (mean ± SD)	3.5 ± 0.2 mm*	4.7 ± 0.2 mm*	1.4 ± 0.08 mm	0.8 ± 0.05 mm
Crestal bone loss (distal) in millimeter (mean ± SD)	3.6 ± 0.08 mm*	4.5 ± 0.3 mm*	1.2 ± 0.07 mm	0.7 ± 0.06 mm

*DM* diabetes mellitus, *SD* standard deviation

*Compared with patients with well-controlled type 2 DM (*P* < 0.01) and normoglycemic individuals (*P* < 0.01)

## Discussion

The reported results support previous studies [10–12, 31] in which an impaired glycemic status (manifested prediabetic and type 2 diabetic patients with chronic hyperglycemia) was described as a significant risk factor of peri-implant mucositis and peri-implantitis. It is noteworthy that the depth of probing and crestal bone resorption on the mesiodistal surfaces of NDIs was nearly 10 times higher in type 2 diabetic patients with chronic hyperglycemia in relation to diabetic patients with optimal glycemic control and non-diabetic individuals. Moreover, clinicoradiographic parameters of peri-implant soft tissue inflammation and CBL were worse type 2 diabetic patients with chronic hyperglycemia in contrast to prediabetic patients. The authors applaud the results reported in the study by Naujokat et al. [13] which showed that under optimal glycemic control, implant therapy is predictable and safe, and the complication rates are comparable with those observed in systemically healthy individuals. From a pathophysiological aspect, studies [17, 22, 32] have shown that AGE-RAGE interactions and their accumulation in the oral and systemic tissues are higher in patients with chronic hyperglycemia. The AGE-RAGE interactions have also been associated with an increased production of inflammatory cytokines (such as TNF-α and IL-1β) in the tissues that in turn worsen gingival inflammation and increase the activity of osteoclasts [33–35]. This is an explanation for the increased peri-implant GI, PD, and CBL among hyperglycemic patients than non-diabetic patients and patients with well-controlled type 2 DM. With reference to the results presented in Table 3, it is noteworthy that in patients with prediabetes and poorly controlled type 2 DM, there was no significant difference in plaque index, and on average, all patients with well-controlled type 2 DM had plaque indices, which were comparable with those among patients without type 2 DM (normoglycemic systemically healthy controls). Indeed, poor plaque control is a risk factor od peri-implant diseases [36]; however, based upon the results obtained, it seems that a state of chronic hyperglycemia is associated with an increased PI in susceptible patient, which in turn makes them more vulnerable to bone loss. Despite having hyperglycemia, HbA1c levels in prediabetic patients (approximately 6%) were significantly lower than those among patients with poorly controlled type 2 DM (approximately 10%). It is therefore anticipated that the accumulation of AGE and production of inflammatory cytokines occur at a slower pace in patients with prediabetes than patients with poorly controlled type 2 DM. This seems to be an explanation for the significantly poorer PI, GI, PD, and CBL in type 2 diabetic patients with chronic hyperglycemia than prediabetic patients. Previous studies [18, 37] on standard diameter (≥ 4 mm) implants have shown that dental implant diabetics can remain functionally and esthetically stable in a manner similar to medially healthy patients provided glycemic levels are strictly controlled. In other words, implant dimensions do not bar the occurrence of peri-implant diseases in patients with an impaired glycemic level. Moreover, according to Oates et al. [37], mechanical implant stability or integration in bone is associated with glycemic control diabetic patients. Although assessment of the mechanical stability of implants in the study groups was out of scope of the present study, it is hypothesized that the mechanical stability of implants in type 2 diabetics with chronic hyperglycemia is poorer in contrast to prediabetic subjects and diabetic individuals having optimal glycemic control. Further studies with long-term follow-up (at least of 5 years) are needed to this hypothesis.

Bone density varies between the maxilla and mandible. The posterior maxilla comprises of type IV (soft bone) bone has a lower bone density compared with the posterior mandible due the presence of trabecular bone [38]. In the present study, NDIs were placed in the posterior maxilla and mandible and it was anticipated that there would be a difference in CBL around NDIs placed in the posterior maxilla and mandible. However, our results showed no statistically significant difference in peri-implant clinicoradiographic parameters around NDIs placed in the maxilla and mandible within each study group (Fig. 1a-d). The only explanation for this result is that nearly 70% of the implants in each group were placed in the mandible. In case there were increased yet statistically comparable numbers of NDIs placed in the maxilla and mandible in each group, a difference in mesiodistal CBL around NDIs in each group could be expected. Further studies are needed in this context.

Based upon evidence from previous studies, a family history of diabetes is more often reported by patients with DM as compared to non-diabetic individuals. The authors of the present study support these results as approximately 50% of patients with prediabetes and poorly controlled type 2 DM stated that their immediate family members (father or mother or both) had DM as compared to non-diabetic patients. However, an interesting finding was that a family history of DM was less often reported by patients with well-controlled type 2 DM in contrast to patients with prediabetes and poorly controlled type 2 DM. It has been reported that an underprivileged education status and poor health literacy and poor are significant risk factors of hyperglycemia among patients with type 2 DM [39, 40]. The authors applaud these studies [39, 40] as the level of attained education was higher in patients with well-controlled type 2 DM (60%) than prediabetic individuals (19%) and patients with poorly controlled type 2 DM (~ 14%). It is worth mentioning that more than 50% of the non-diabetic controls and patients with well-controlled type 2 DM reported to brush their teeth twice daily compared with individuals with prediabetes and poorly controlled type 2 DM. It is speculated that an advanced education status and health literacy played a positive role in compelling these individuals to take care of their oral hygiene status compared with patients with poorly controlled type 2 DM and prediabetes. The authors recommended that community-based health awareness programs should routinely be conducted to educate the general public about the detrimental effects of hyperglycemia on overall health and the benefits of routine oral hygiene maintenance toward the attainment of a superior quality of life.

One limitation of the present study is that most of the implants were placed in the posterior mandible. Moreover, tobacco smokers and individuals using ST products were excluded. Furthermore, the mechanical stability of NDIs was not assessed within and among the study groups. We hypothesize that the severity of peri-implant diseases is worse in hyperglycemic smokers and ST chewers compared with non-diabetic individuals not using any form of tobacco product. Further studies are needed to test this hypothesis.

## Conclusion

Chronic hyperglycemia increases the risk of peri-implant diseases around NDIs.

## Supplementary information

**Additional file 1.** STROBE Statement—checklist of items that should be included in reports of observational studies.

## Data Availability

The datasets related to the current study are available from the corresponding author on reasonable request.

## Abbreviations

AGEs: Advanced glycation endproducts
CBL: Crestal bone loss
DM: Diabetes mellitus
GI: Gingival index
IAJ: Implant abutment junction
IL: Interleukin
NDIs: Narrow diameter implants
PD: Probing depth
PI: Plaque index
RAGE: Receptors for advanced glycation endproducts
ST: Smokeless tobacco
TNF-alpha: Tumor necrosis factor alpha
